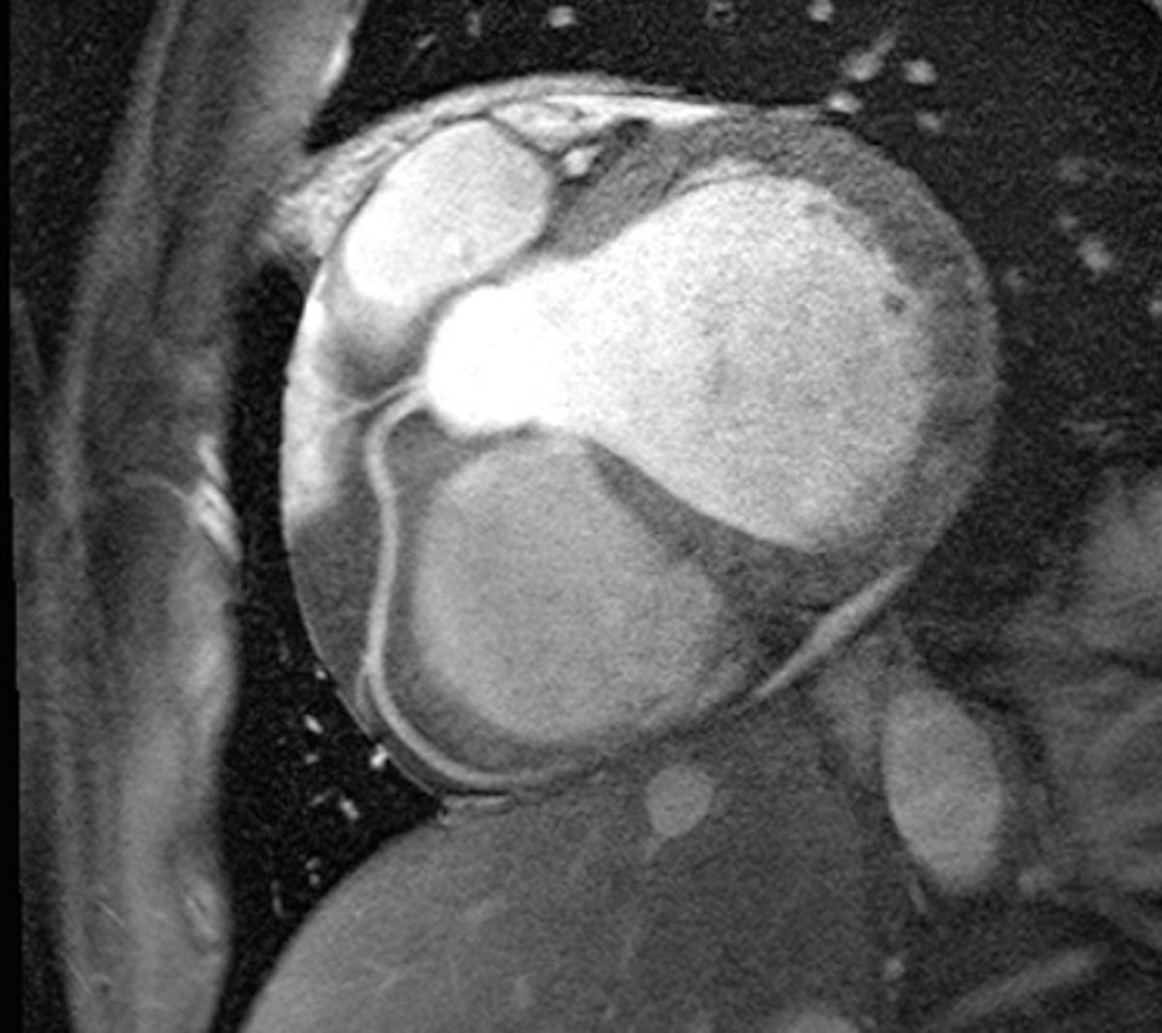# Value of a hybrid PET/MRI in the assessment of cardiac viability

**DOI:** 10.1186/1532-429X-14-S1-P80

**Published:** 2012-02-01

**Authors:** David Carballo, René Nkoulou, Gabriella Vincenti, Alessandra Quercioli, Susanne Heinzer, Dominique Didier, Matthias Stuber, Thomas Schindler, Osman Ratib, Jean Vallee

**Affiliations:** 1 Geneva University Hospital, Geneva, Switzerland; 2 Lausanne University Hospital, Lausanne, Switzerland

## Summary

Hybrid PET/MRI imaging techniques are become more readily available, and its role in viability assessment appears promising.

## Background

We evaluated the potential for hybrid PET/MRI devices to provide integrated metabolic, functional and anatomic characterization of patients with fixed hypoperfusion on cardiac SPECT.

## Methods

A pilot study of eleven patients (6 fixed hypoperfusion on cardiac SPECT and 5 healthy volunteers) performed an imaging study using a hybrid PET/MRI (Philips). Viability assessed by 18F-FDG was performed in diseased patients along with MRI anatomic, functional and viability assessment as determined by transmural extent of late gadolinium enhancement (LGE). A reassessment by conventional PET/CT was carried out within 30 minutes. Non-contrast right coronary artery (RCA) targeted and whole heart 3D coronary angio-MRI using ECG-gating and respiratory navigator was performed in healthy volunteers with reconstruction performed using MPR and volume rendering. The extent of metabolic defect (MD) using PET/MRI and PET/CT were compared in patients and coronary territories (LAD, CX, RCA). Assessibility of coronary lumen was judged as good, sub-optimal or non-assessable using a 16-segments coronary model.

## Results

Direct comparison of myocardial viability as assessed by MRI LGE and Pet metabolism revealed consistent results in 4 of the 6 diseased patients, and discordant results in the other two. Of note, one discordant result was potentially due to suboptimal attenuation correction in the case of a hypertrophied left ventricle. Metabolic assessment was successful in all patients with MD being 19.2% vs 18.3% using PET/MRI and PET/CT, respectively (P= ns). The MD was 10.2%, 6%, and 3% vs 9.3%, 6% and 3% for LAD, CX and RCA territories, respectively (P= ns). Coronary angio-MRI was successful in all volunteers with 66 coronary segments visualized overall. The RCA was fully visualized in 4/5 volunteers and the left coronary arteries in 4/5 volunteers. Assessibility in visualized segments was good, sub-optimal and non assessable in 88%, 2% and 10%, respectively.

## Conclusions

Hybrid PET/MRI does not compromise image quality and in addition provides clinically significant results for myocardial viability assessment.

## Funding

Institutional Funding, Geneva University Hospitals.

**Figure 1:**
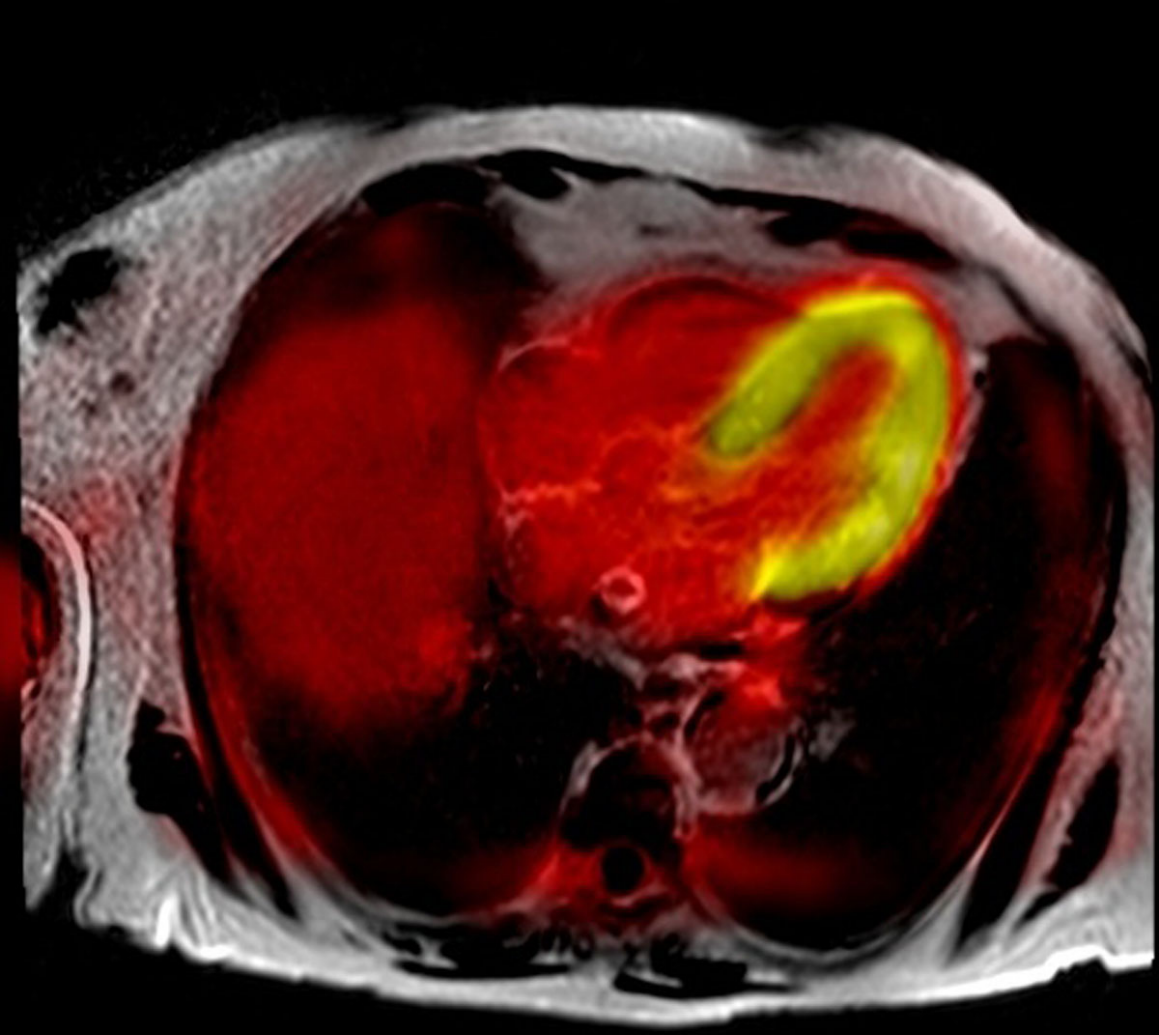


**Figure 2:**